# Effects of Oleacein on High-Fat Diet-Dependent Steatosis, Weight Gain, and Insulin Resistance in Mice

**DOI:** 10.3389/fendo.2018.00116

**Published:** 2018-03-19

**Authors:** Giovanni Enrico Lombardo, Saverio Massimo Lepore, Valeria Maria Morittu, Biagio Arcidiacono, Carmela Colica, Antonio Procopio, Valentina Maggisano, Stefania Bulotta, Nicola Costa, Chiara Mignogna, Domenico Britti, Antonio Brunetti, Diego Russo, Marilena Celano

**Affiliations:** ^1^Department of Health Sciences, University “Magna Græcia” of Catanzaro, Catanzaro, Italy; ^2^CNR, IBFM UOS of Germaneto, University “Magna Græcia” of Catanzaro, Catanzaro, Italy; ^3^Interdepartmental Service Center, University “Magna Græcia” of Catanzaro, Catanzaro, Italy

**Keywords:** oleacein, insulin resistance, obesity, liver steatosis, cholesterol

## Abstract

Many reports indicate that the protective action of nutraceuticals in the Mediterranean diet, against metabolic and cardiovascular diseases, can be attributed to the action of polyphenolic components of extra-virgin olive oil (EVOO). Here, we evaluated the protective effects of oleacein, one of the most abundant secoiridoids in EVOO, on the damages/metabolic alterations caused by high-fat diet (HFD) in male C57BL/6JolaHsd mice. After 5 weeks of treatment with 20 mg/kg of oleacein, body weight, glycemia, insulinemia, serum lipids, and histologic examination of liver tissue indicated a protective action of oleacein against abdominal fat accumulation, weight gain, and liver steatosis, with improvement of insulin-dependent glucose and lipid metabolism. Both serum parameters and hepatic histologic examination were altered in mice fed with HFD. By contrast, in the animals that received oleacein, plasma glucose, cholesterol and triglyceride serum levels, and liver histology were similar to controls fed with normocaloric diet. In addition, protein levels of FAS, SREBP-1, and phospho-ERK in liver were positively modulated by oleacein, indicating an improvement in liver insulin sensitivity. In a group of obese mice, treatment with oleacein determined a light, but still significant reduction of the increase in body weight, mainly due to lesser liver steatosis enlargement, associated with reduced levels of SREBP-1 and phospho-ERK and lower levels of total serum cholesterol; in these animals, altered plasma glucose and triglyceride serum levels were not reverted by oleacein. These results indicate that HFD-related hepatic insulin resistance may be partially prevented by oral administration of oleacein, suggesting a protective role of this nutraceutical against diet-dependent metabolic alterations. Additional studies are necessary to check whether oleacein can be used as an adjuvant to improve insulin sensitivity in humans.

## Introduction

In the last few decades, a higher frequency of obesity, type 2 diabetes mellitus, and metabolic syndrome has been registered, as a result of various dietary and evolutionary changes and modernizing influences (1, 2). Results suggest that insulin resistance could be considered a common pathogenic factor for all these conditions, which may be prevented and even counteracted by a healthy lifestyle that includes a correct diet (3–6). In this regard, an adjuvant action may be played by some nutraceuticals of proven beneficial effects, such as the polyphenols found in the extra-virgin olive oil (EVOO), a peculiar component of Mediterranean diet, which has been proposed by many studies as a protective factor against several diseases (7). In particular, the pro-active ingredient oleuropein and its derivative hydroxytyrosol have been widely investigated, demonstrating, even when tested as single compounds, many beneficial effects, either *in vitro* or *in vivo*, in experimental animal models (8–10). However, the amount of oleuropein in the EVOO is too low to account for a significant role when assumed in the EVOO. Actually, the endogenous β-glucosidase liberated during the olive oil extraction process, hydrolyzes oleuropein giving rise to a series of degradation products, all of which are less hydrophilic than the original natural secoiridoids, and therefore more soluble in the oily matrix extracted from the drupes. Thus, EVOO is scarce in oleuropein and much more abundant in its degradation product, oleacein (11–13), whose lipophilicity suggests that this compound may survive to the acidic conditions of stomach and be available for absorption into the systemic circulation, where it may reach considerable concentration (from 1 to 18 µM), thereby making it one of the effectors of the nutritional and beneficial effects of EVOO (14, 15).

At present, there are no data on the effects of oleacein on glucose/lipid metabolism when administered in association with a high-fat diet (HFD). In the present study, we analyzed the effects of oral administration of oleacein in C57BL/6JOlaHsd mice fed with a HFD. Measure of body weight, glycemia, insulinemia, serum lipids, and histologic examination of liver tissue were performed after 5 weeks of oleacein treatment. The same measurements were also performed after an additional 8 weeks’ treatment in animals that had become overweight. A mechanistic explanation of oleacein effects was investigated in liver tissue as well.

## Materials and Methods

### Chemicals

Oleuropein was extracted as reported previously (16) from olive leaves of Coratina cultivar of *Olea europaea*. The purity was determined by RP-HPLC and HRMS-ESI and compared with data reported in the literature (16). Oleacein is the dialdehydic form of oleuropein’s aglycone [2-(3,4-hydroxyphenyl) ethyl (3S,4E)-4-formyl-3-(2-oxoethyl) hex-4-enoate]. Oleacein was obtained as described elsewhere, *via* one-step semi-synthesis under microwave assisted aqueous Krapcho decarbomethoxylation of oleuropein (14).

### Ethics Statement

All experiments were performed in accordance with the guidelines of the Italian (D.M. 116/92) and ECC regulations (O.J. of E.C.L 358/1 12/18/1986), after approval of the experimental protocol by the local Ethic Committee of the University “Magna Græcia” of Catanzaro. Every precaution was taken to minimize stress and the number of animals used in each series of experiments.

### Animals and Study Design

Five-week-old male C57BL/6JOlaHsd mice (*n* = 28), normocaloric diet (TD. 2018), and HFD (TD. 06414) were purchased from Envigo RMS S.r.l. Mice were maintained in 12-h light/dark cycle at 21 ± 1°C and 50 ± 5% humidity with free access to water and food *ad libitum*. Initially, 28 animals were randomized in four groups: mice fed with normocaloric diet (NCD, *n* = 8), mice fed with HFD (HFD, *n* = 12), and mice fed with HFD with a daily oral gavage of 20 mg/kg of oleacein (HFD-OLEAC, *n* = 4) or oleuropein (HFD-OLE, *n* = 4) for 5 weeks. In the second set of experiments, eight mice from the initial HFD group were divided into two groups and continued to receive the HFD without (HFD group, *n* = 4) or with a daily oral gavage of 20 mg/kg of oleacein (HFD-OLEAC group, *n* = 4) for additional 8 weeks. At the end of treatments (5 and 13 weeks in the two sets of experiments, respectively), animals were sacrified through cervical dislocation after 12 h overnight fasting. The whole liver, abdominal fat, heart, kidney, lung, and spleen were excised, weighted, and stored in liquid nitrogen, or washed in normal saline solution and immediately fixed in 10% neutral buffered formalin (Sigma-Aldrich S.r.l.). Body weight, food, and energy intake were recorded at weekly interval (17). In particular, body weight was analyzed as repeated measures. Insulin tolerance test (ITT) was performed in NCD, HFD, and HFD-OLEAC mice, as previously described (18). Insulin (1 U/kg body weight) was injected intraperitoneally into 12 h-fasted animals, and blood glucose levels were measured after 0, 15, 30, 60, and 90 min using an automatic glucometer (Glucocard Menarini).

### Biochemical Analysis

Blood samples were collected after 12 h overnight fasting and glucose levels were then measured using a glucometer. Serum was obtained by centrifugation at 1,700 *g* for 10 min at room temperature and stored at −80°C until use. Total cholesterol, high-density lipoprotein cholesterol, low-density lipoprotein cholesterol, triglycerides (Abcam), and fasting insulin (EMD Millipore Corporation) were measured using commercial kits, according to the manufacturer’s instructions, as previously described (19, 20). Approximate insulin resistance was calculated using the homeostasis model assessment [homeostatic model assessment of insulin resistance (HOMA-IR)], using the following formula: [glucose (mg/dl) × insulin (mU/l)/405] (21).

### Protein Extraction and Western Blotting

Total proteins were extracted from liver tissue, homogenizing the samples in modified RIPA buffer [50 mM Tris–HCl pH 7.5, 150 mM NaCl, 1% Triton, 0.25% sodium deoxycholate, 10 mM sodium pyrophosphate, 1 mM sodium orthovanadate, 1 mM sodium fluorure (Sigma-Aldrich), and protease inhibitor cocktail (Roche Diagnostics GmbH)], and then centrifuging the supernatant at 10,000 *g* for 10 min at 4°C, twice (18). 15 µg of total protein was separated through SDS-polyacrylamide gel electrophoresis (run on 7 and 12%), as previously described (22, 23). Protein expression was measured using the following specific antibodies: anti-sterol regulatory element-binding transcription factor 1 (SREBP-1; 1:500); anti-extracellular regulated MAP kinase (ERK; 1:1,000) and anti-phospho-ERK (Thr202/Tyr204 p-ERK; 1:500) (Santa Cruz Biotechnology); anti-fatty acid synthase (FAS; 1:1,000) (Cell Signaling Technology); and anti-GAPDH (1:40,000) (Thermo Fisher Scientific Inc.). Western blot (WB) detection system ECL Plus (Perkin Elmer) was used to visualize the immune complexes (24).

### Histopathological Study

Hematoxylin and eosin staining were performed according to the standard methods for histological assessment under light microscopy for toxicological analysis. Steatosis evaluation was performed by count of the percentage of vacuolated cells. A numerical score was assigned to each section on the basis of the percentage of vacuolated cells calculated for each mouse liver: 1 = 1–25%; 2 = 30–65%; 3 = >70%.

### Statistical Analysis

Data are expressed as mean ± SD and were analyzed using GraphPad Prism version 5.0 statistical software (GraphPad Software Inc.). Significant differences were analyzed by one-way analysis of variance followed by Tukey multiple comparison test (25). *p*-Values <0.05 were considered statistically significant.

## Results

### Effects of Oleacein on Body Weight and Biochemical Parameters

**Figure 1:**
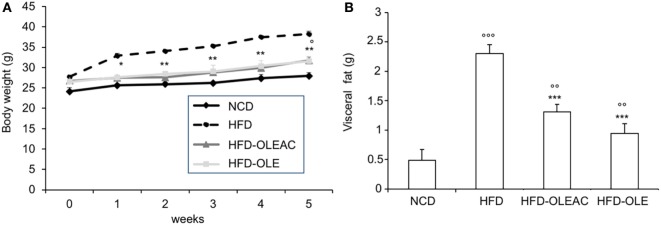
Effects of oleacein on body and visceral fat weight in mice fed with high-fat diet (HFD). Body and visceral fat weight were measured in mice fed with normocaloric diet (NCD), HFD, and HFD with oleacein (HFD-OLEAC) or oleuropein (HFD-OLE) during **(A)**, and at the end **(B)** of 5 weeks treatment. Values are expressed as mean ± SD. *p < 0.05, **p < 0.01, ***p < 0.001 vs HFD; ^°^*p* < 0.05, ^°°^*p* < 0.01, ^°°°^*p* < 0.001 vs NCD.

**Figure 2:**
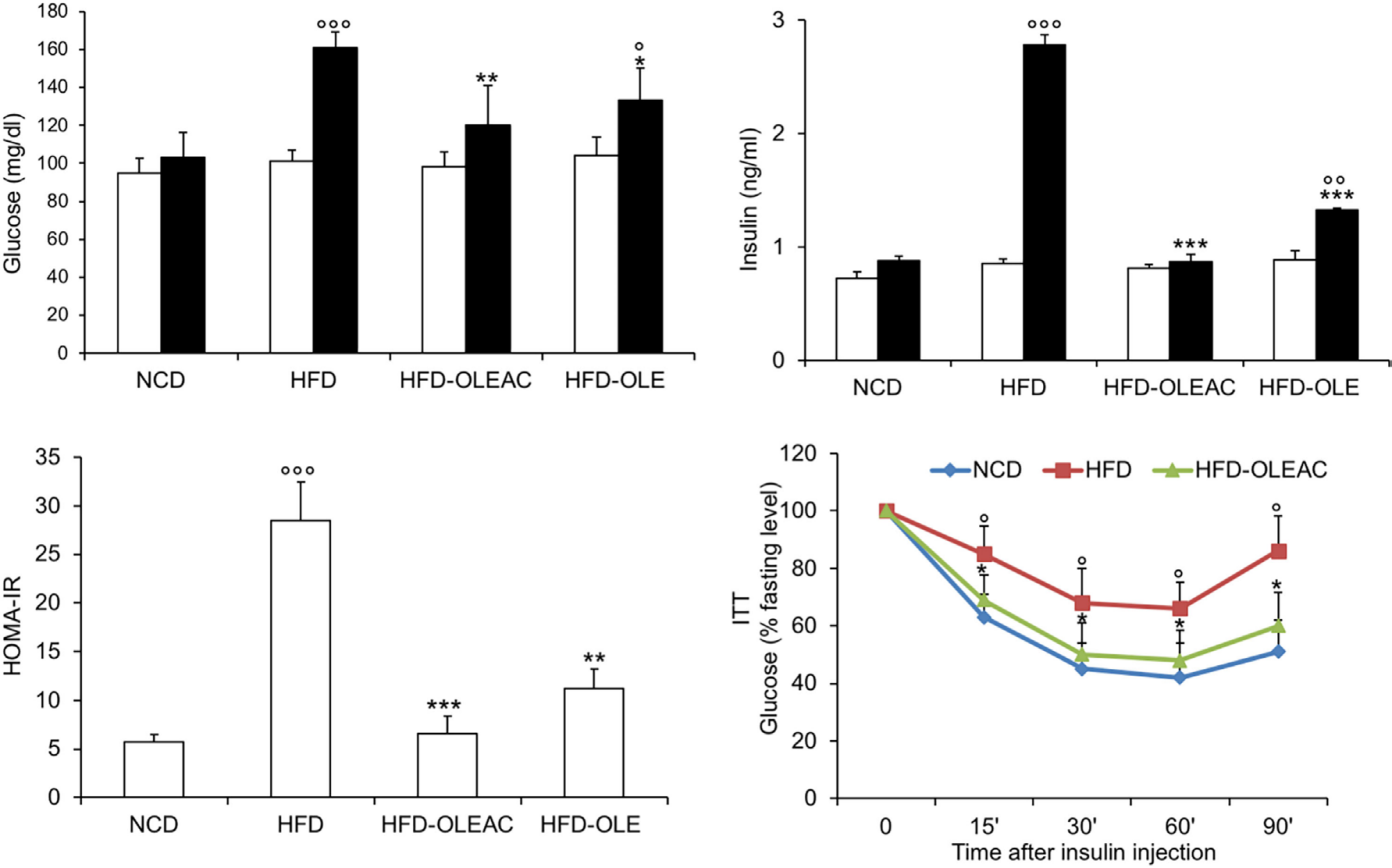
Effects of oleacein on insulin resistance. Fasting plasma glucose and insulin levels were measured at beginning (white bars) and at the end (black bars) of 5 weeks of treatment with oleacein (HFD-OLEAC) or oleuropein (HFD-OLE). Homeostatic model assessment of insulin resistance (HOMA-IR) was calculated thereafter. Insulin tolerance test (ITT) in NCD, high-fat diet (HFD), and HFD-OLEAC mice (*n* = 4–6 per group). Values are expressed as mean ± SD. *p < 0.05, **p < 0.01, ***p < 0.001 vs HFD group. ^°^*p* < 005, ^°°^*p* < 0.01, ^°°°^*p* < 0.001 vs NCD.

**Figure 3:**
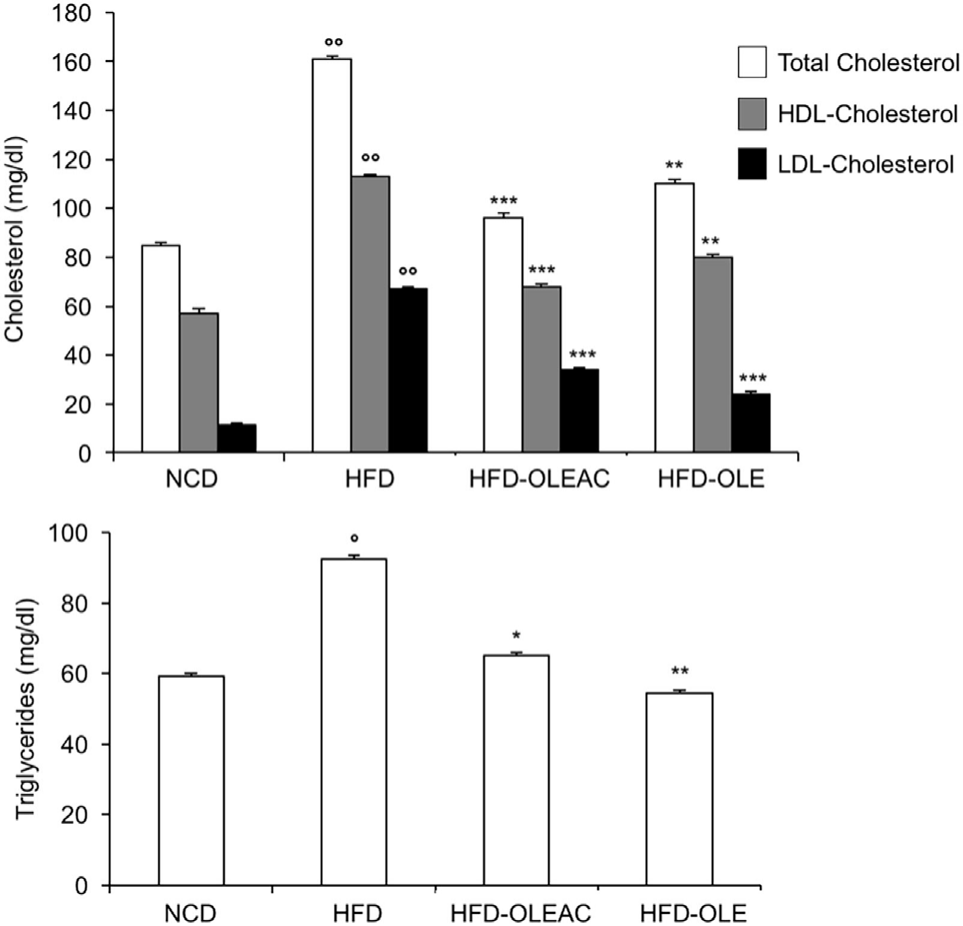
Biochemical parameters. Blood samples were collected as indicated in the Section “Materials and Methods.” After 5 weeks of feeding with high-fat diet (HFD), total cholesterol, low-density lipoprotein cholesterol (LDL-C), and high-density lipoprotein cholesterol (HDL-C) were measured in the indicated groups of mice, together with triglyceride levels. Values are expressed as mean ± SD. *p < 0.05, **p < 0.01, ***p < 0.001 vs HFD. ^°^*p* < 0.05, ^°°^*p* < 0.001 vs NCD.

The effects of oleacein or oleuropein (20 mg/kg each) were first analyzed in HFD fed mice, following a 5-week treatment period. An increase in body weight was observed in HFD mice as compared to the NCD group, and this increase was primarily due to visceral fat accumulation (Figures 1A,B). Body weight in HFD mice was significantly higher than all other groups (Figure 1A), thereby indicating a preventive action of oleacein (and its precursor oleuropein, used herein as a positive control) on body weight enhancement. No changes were noted in food and energy intake, or water intake when oleacein-HFD treated mice were compared weekly with oleacein-untreated HFD mice under standard housing during this study (Table 1). Also, during the experimental period, no signs of suffering or behavioral changes were noted among animal groups. The biochemical parameters analyzed after this time period showed that, in the HFD group of animals, a typical picture of HFD-induced metabolic alterations was present, including hyperglycemia and hyperinsulinemia (Figure 2), with an increase in serum levels of total cholesterol and triglycerides (Figure 3). Administration of oleacein counteracted these effects, inducing changes in all these parameters, and resulting even stronger than oleuropein in reducing insulin levels (Figures 2 and 3), and in amelioreting insulin sensitivity, as documented by both HOMA-IR and ITT (Figure 2).

**Table 1:** Effects of oleacein on final food and energy intake after 5 weeks high-fat diet (HFD).

	NCD	HFD	HFD-OLEAC	HFD-OLE
Food intake (g/week)	19.6 ± 0.5	17.8 ± 0.6	17.6 ± 0.4	17.0 ± 0.1
Energy intake (kcal/week)	67.1 ± 4.0	90.6 ± 3.4	89.7 ± 1.0	86.1 ± 1.4

*Values are expressed as mean ± SD. HFD, HFD-OLEAC, and HFD-OLE indicate mice fed with HFD, or HFD and oleacein or oleuropein, respectively. NCD indicates the animals fed with normocaloric diet*.

### Effects of Oleacein on Liver Morphology and Molecular Targets

**Figure 4:**
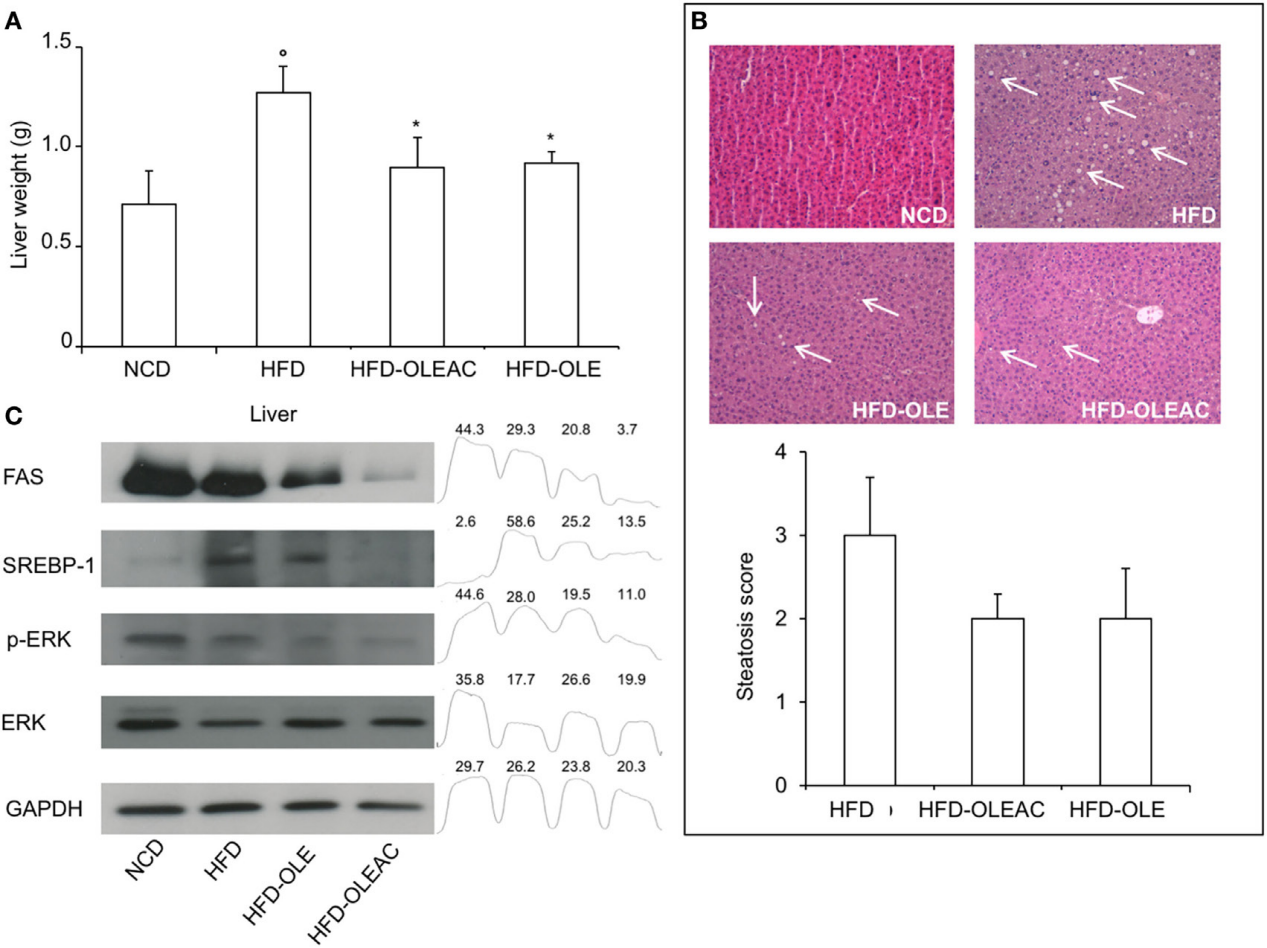
Effects of oleacein on liver. **(A)** Liver weight was significantly reduced in mice fed with high-fat diet (HFD) supplemented with oleacein (HFD-OLEAC) or oleuropein (HFD-OLE) after 5 weeks treatment. Values are mean ± SD. *p < 0.05 compared with HFD. ^°^*p* < 0.01 vs NCD. **(B)** Hematoxylin and eosin staining of liver sections (20× magnification). Liver from HFD fed mice was rich in fat vacuoles (indicated by the arrows). For steatosis evaluation, a numerical score was assigned to each section, on the basis of the percentage of vacuolated cells as calculated for each mouse liver: 1 = 1–25%, 2 = 30–65%, 3 = >70%. **(C)** Reduced levels of FAS, SREBP-1, ERK, and p-ERK were detected by Western blot (WB) analysis in liver protein extracts from oleacein- or oleuropein-treated animals (*n* = 3–4 per group), as compared to protein levels from mice fed with NCD or HFD (each, *n* = 4). GAPDH, control of protein loading. Densitometric analysis from a representative WB (representative of three separate assays) is shown. Numbers on the peaks are the size of the corresponding slot as a percentage of the total size of the four slots in each condition.

**Figure 5:**
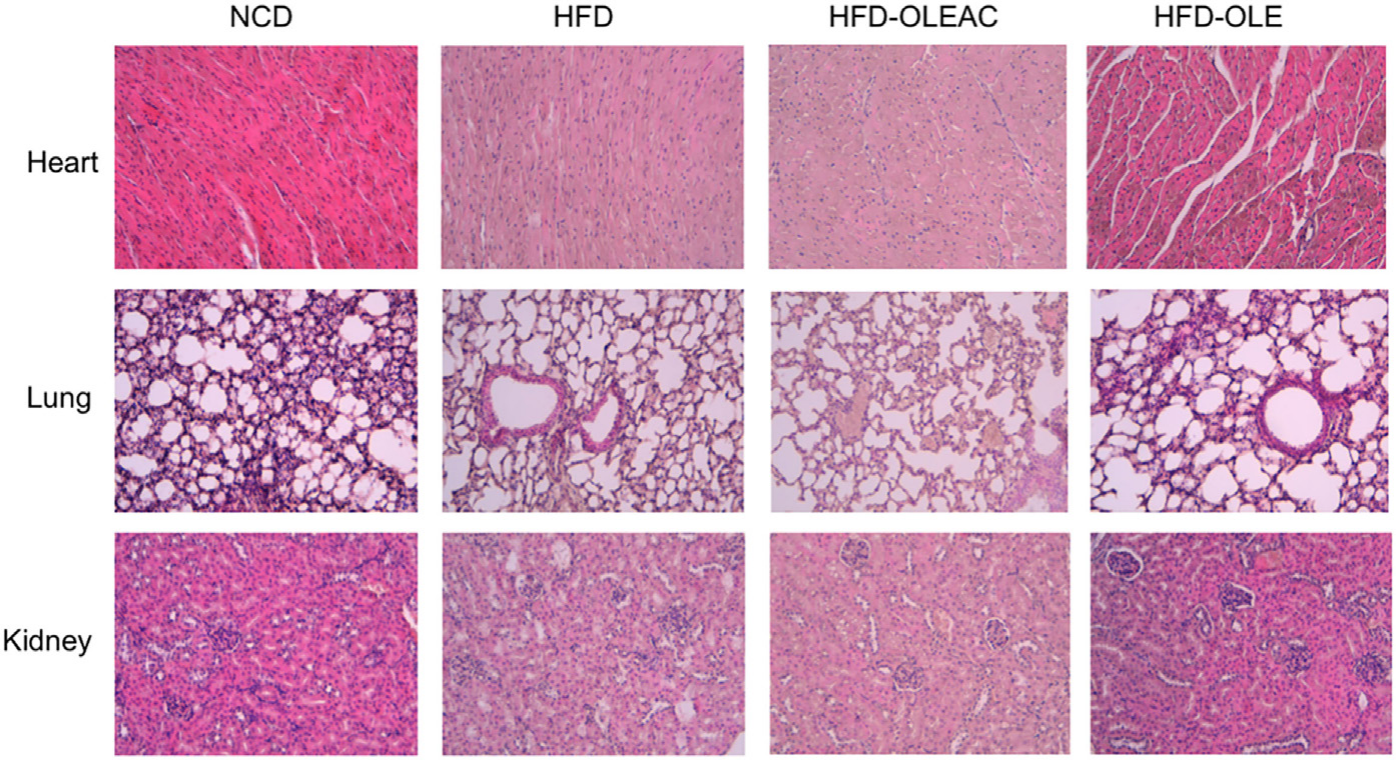
Histologic examination of heart, lung, and kidney sections. No appreciable histological differences were detected at these levels, among the four groups of mice, at the end of the 5 weeks treatment period with either oleacein (HFD-OLEAC) or oleuropein (HFD-OLE).

Treatment with oleacein (or oleuropein) prevented the hepatic enlargement, as evident by the liver weight values (Figure 4A), and the development of HFD-dependent hepatic steatosis, as proven by histologic examinations and quantitative evaluation of steatosis (Figure 4B). WB analyses with liver protein extracts showed a reduction in the expression of SREBP-1 and FAS, as well as in p-ERK in the HFD group of mice treated with either oleacein or oleuropein, suggesting a role for these enzymes/molecular targets as potential mediators of the effects of these compounds (Figure 4C). No signs of toxicity appeared in lung, kidney, and heart tissues of animals treated with oleacein or oleuropein (Figure 5).

### Effects of Oleacein on Mice With Established Obesity

**Figure 6:**
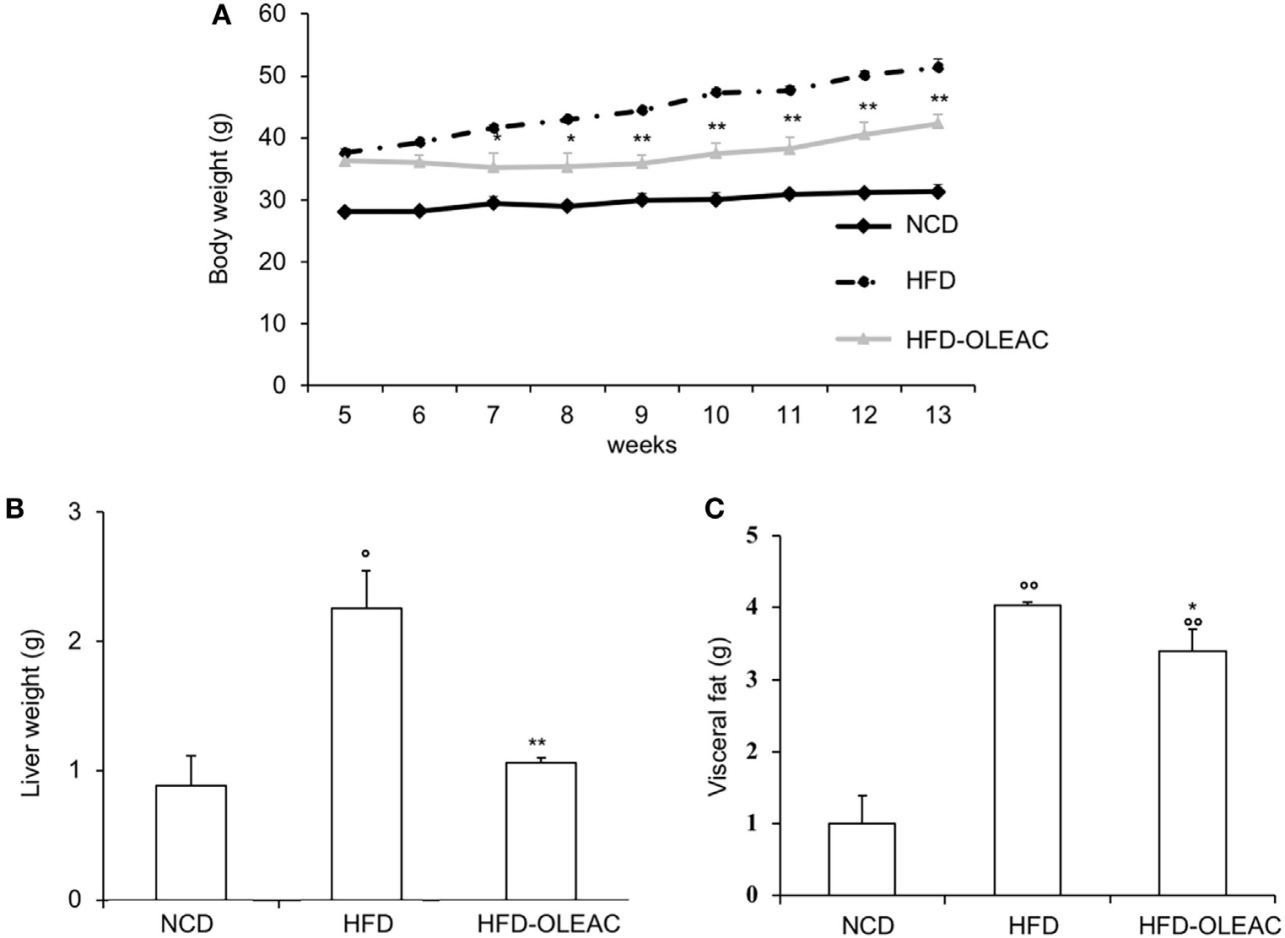
Effects of oleacein on body weight, visceral fat, and liver weight in obese mice fed with high-fat diet (HFD). Body weight **(A)**, liver weight **(B)**, and visceral fat **(C)** were measured in obese mice fed with HFD. Values are expressed as mean ± SD. *p < 0.05, **p < 0.01 vs HFD. ^°^*p* < 0.05, ^°°^*p* < 0.01 vs mice fed with NCD. HFD-OLEAC, mice treated with oleacein.

**Figure 7:**
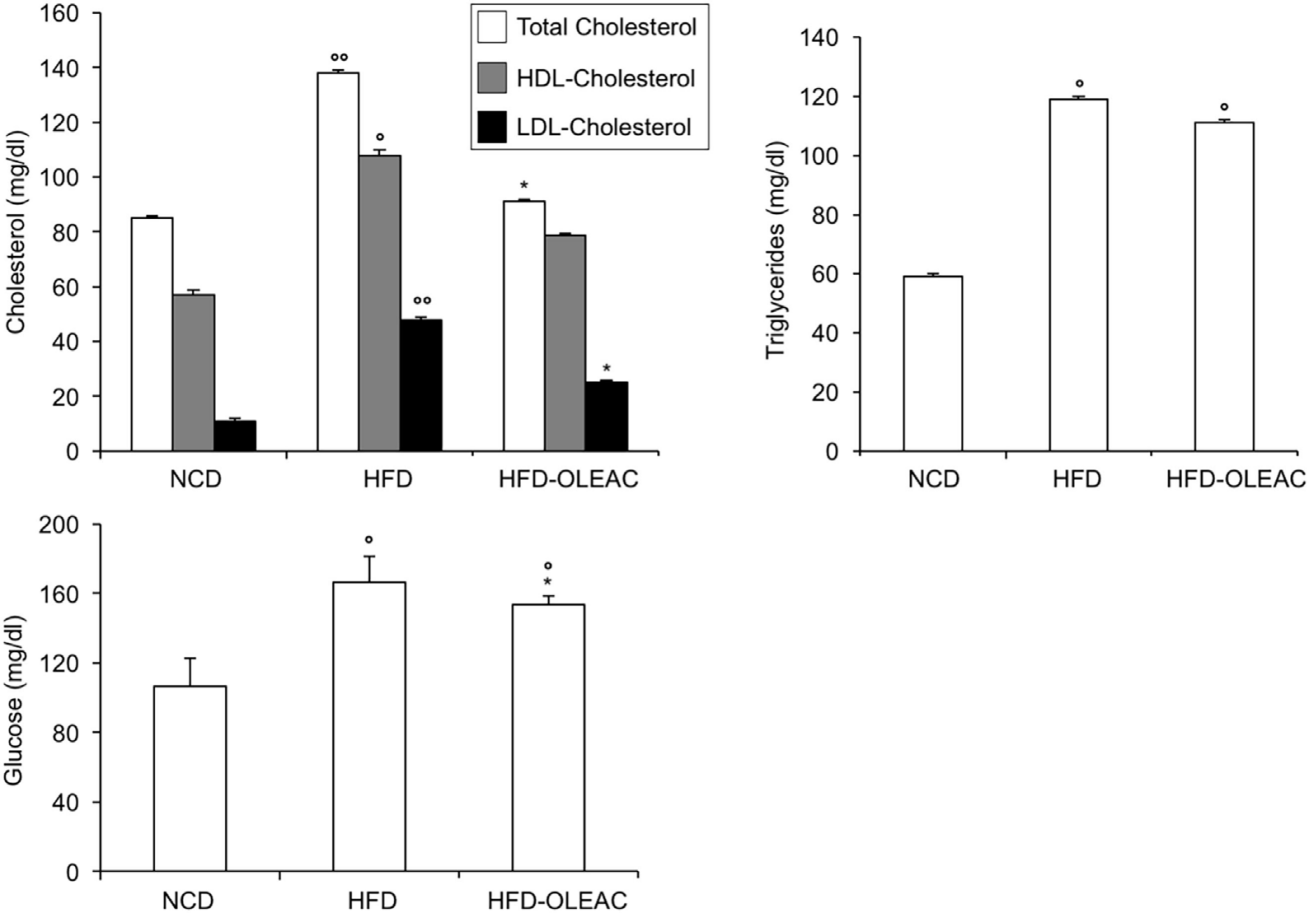
Effects of oleacein on lipid serum levels in obese mice fed with high-fat diet (HFD). Blood samples were collected as indicated in the Section “Materials and Methods.” After 13 weeks of feeding with HFD, total cholesterol, high-density lipoprotein cholesterol (HDL-C), and low-density lipoprotein cholesterol (LDL-C) were measured together with triglyceride and plasma glucose levels. Values are expressed as mean ± SD. *p < 0.001 vs mice fed with HFD. ^°^*p* < 0.05, ^°°^*p* < 0.01 vs mice fed with NCD.

**Figure 8:**
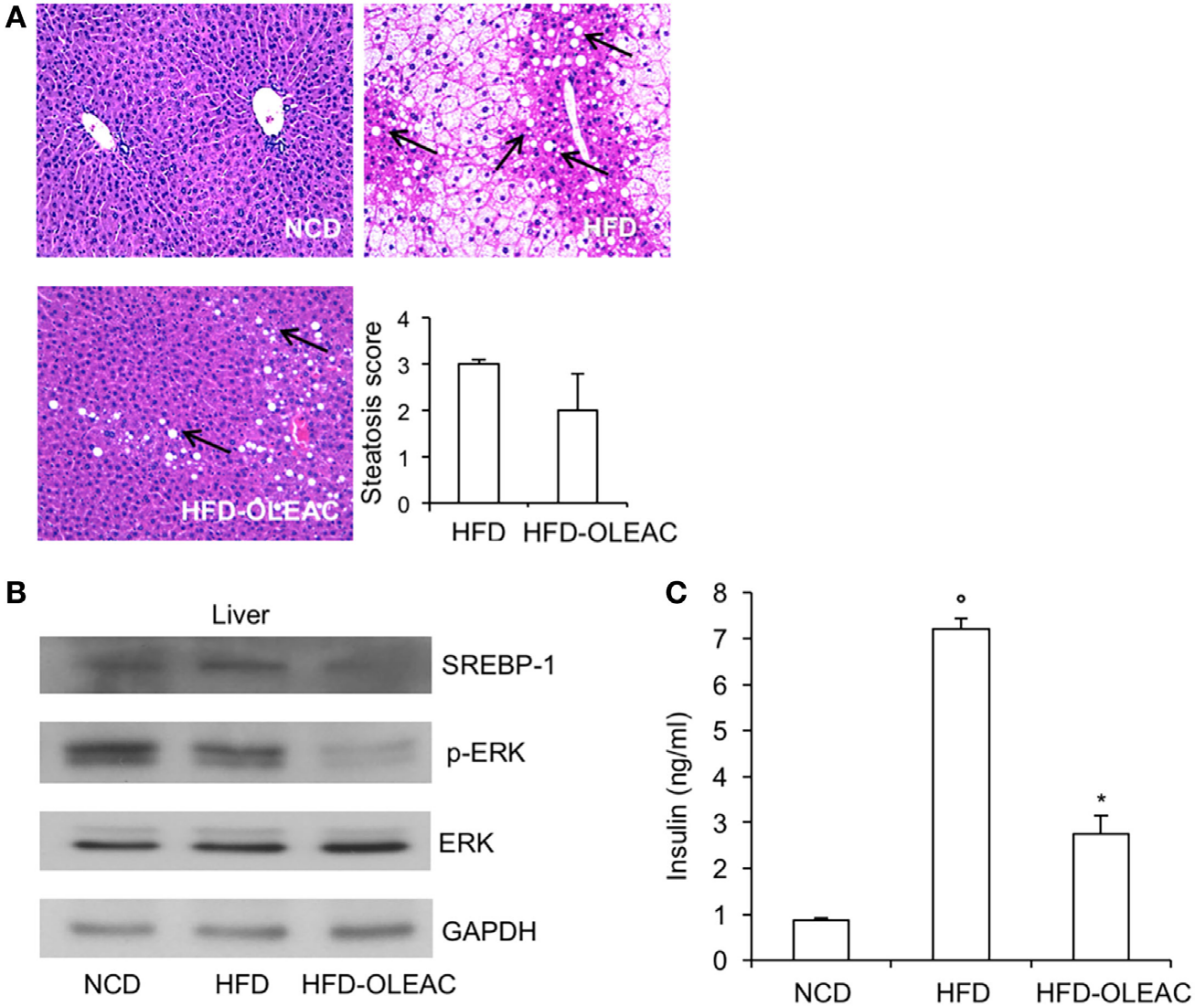
Effects of oleacein on insulin levels, liver steatosis, and Western blot analyses of SREBP-1 and ERK. **(A)** Hematoxylin and eosin staining of liver sections (20× magnification). Liver of obese mice fed with high-fat diet (HFD) was rich in fat vacuoles (indicated by the arrows). For steatosis evaluation, a numerical score was assigned to each section, on the basis of the percentage of vacuolated cells as calculated for each mouse liver: 1 = 1–25%, 2 = 30–65%, 3 = >70%. **(B)** Protein expression levels of SREBP-1, ERK, and p-ERK were detected in liver from oleacein-treated and untreated mice fed with HFD and mice fed with NCD. GAPDH, control of protein loading. **(C)** Blood samples were collected as indicated in the Section “Materials and Methods.” Insulin levels were measured after 13 weeks of feeding with HFD with and without oleacein. Values are expressed as mean ± SD. *p < 0.05 vs mice fed with HFD. ^°^*p* < 0.05 vs mice fed with NCD.

In a second set of experiments, we investigated the effects of oleacein on already overweight mice. To this end, eight animals from the HFD group (mean weight of 37.6 ± 0.6 g) continued to be fed with HFD while receiving or not receiving the same dose of oleacein for further 8 weeks. Once again, a reduced increase in body weight was observed in oleacein-treated mice (Figure 6A), mainly due to a lesser increase in liver tissue (Figure 6B), and, to a lesser extend, to abdominal fat (Figure 6C). As shown in Figure 7, only total cholesterol levels were significantly corrected by oleacein, whereas levels of tryglicerides were not affected, and glycemia was only slightly modified. Also, oleacein reduced lipid deposits in the liver (Figure 8A), and this reduction paralleled the decrease in p-ERK and SREBP-1 protein expression (Figure 8B), a condition that could contribute to ameliorating hyperinsulinemia in HFD oleacein-treated mice (Figure 8C).

Therefore, altogether, these data indicate that the improvement in insulin sensitivity in oleacein-treated mice, together with changes in some hepatic molecular targets of insulin, along with the reduction in visceral fat, may provide, at least in part, a possible explanation as to how oleacein can prevent weight gain in HFD fed mice. Studies for a more detailed mechanistic explanation in this direction are in progress.

## Discussion

The negative influence of excessive body fat on insulin action and hepatic function, leading to peripheral insulin resistance, has been widely demonstrated, indicating that a direct link does exist between obesity, type 2 diabetes, hypertension, non-alcoholic hepatic steatosis, obstructive sleep apnea, and atherosclerosis (26, 27). Thus, in all these conditions, changes in the lifestyle, including dietary and nutritional changes, still represent the first-line therapeutic approaches (26, 27). A wide number of studies have been performed on the effects of nutraceutical compounds or other food components. Among these, the well-known protective properties of EVOO-related compounds, key components of Mediterranean diet, have encouraged the investigation of mixtures of phenolic extracts from olive drupes or leaves or single compounds, such as oleuropein and its derivate hydroxytyrosol, both *in vitro* and *in vivo* experimental model systems (8–10).

We have previously reported the beneficial properties of *in vivo* administration of oleuropein, showing its protective effect against the high-fat cafeteria diet-induced body weight gain and hepatic steatosis, and a lower increase of cholesterol and glucose levels with improvement in peripheral insulin sensitivity (17). Prevention of HFD-induced adiposity and the hepatoprotective action of oleuropein in mice were also reported in other studies, in which oleuropein supplementation was able to modulate the expression of a variety of liver and adipose specific genes involved in the oxidative stress, lipid detoxification, and inflammation (28–31).

In the present study, we tested the effects of oleacein as obtained by using a simple and environmental friendly method, starting from the easily available natural oleuropein (16). This compound, which is the real oleuropein derivative present in EVOO, displays antioxidant and antiflogistic properties, when used at a concentration comparable with that found in EVOO (15, 32). Here, we show that oral administration of 20 mg/kg/day of oleacein exerts a protective, beneficial effect on several HFD-related metabolic alterations, including a reduction in the body weight increase due to minor increment of fat in the abdominal visceral adipose tissue, as well as a significant decrease of macroscopic and microscopic steatosis. In addition, the association of oleacein treatment with lower levels of serum lipids, and partial correction of high plasma glucose levels, without increasing plasma insulin levels, is compatible with a role for oleacein in improving insulin sensitivity, which was confirmed by ITT. Our opinion in this regard is that oleacein, by ameliorating insulin sensitivity and reducing circulating insulin levels, can prevent metabolism dysfunction and weight gain. This explanation is supported by the finding that visceral fat (strongly linked to insulin resistance and metabolic disease) was significantly reduced in oleacein-HFD-treated mice, as compared to oleacein-untreated HFD mice. As an endocrine organ, adipose tissue produces a variety of local and systemic factors that regulate food intake, insulin sensitivity, and energy homeostasis. By disrupting the proper balance and function of these factors, fat expansion promotes insulin resistance (33). Therefore, based on the data obtained in our study, the mechanism for preventing weight gain in oleacein-treated mice may be related to the amelioration of insulin resistance and the prevention of metabolic dysfunction. Weight loss related to improved insulin resistance and insulin sensitivity has been reported in diet-induced obese rats in which changes in the size of adipocytes and reduction in fat tissue were observed (34). An alternative, unexplored hypothesis, compatible with our findings, is that oleacein, like metformin (35), the first-line drug for treatment of insulin-resistant diabetes, may act by increasing energy expenditure, thus preventing weight gain. As a limit of this study, we currently cannot perform calorimetric measurements, so that future investigation is needed to validate this possibility.

Furthermore, for the first time in this study, we have identified some of the molecular constituents that appear to be involved in oleacein action, including some known regulators of hepatic lipid metabolism. Indeed, the levels of the transcriptional activator SREBP-1 and its target FAS, key factors regulating the relative contribution of liver and adipose tissue to lipogenesis (36–42), were increased in the liver of HFD mice and were reduced significantly following treatment of animals with oleacein. Protection from liver damage correlated also with the inhibition, in liver tissue, of the p-ERK, a condition that has been recently associated with amelioration of insulin resistance in rat liver (43). Some of these studies also reported the association of such hepatic markers with reduction of the weight gain determined by phenolic compounds (30, 31).

Our findings from our second set of experiments, in which oleacein was administrated in already obese animals, showed that oleacein was able to counteract only partially some of the adverse effects of HFD. Among them, a reduced increase in body weight and liver mass, with reduced lipid infarction of the liver. Additional beneficial effects included the decrease of cholesterol levels, whereas hyperglycemia and triglyceridemia were not fully corrected, thus indicating that no real curative effect of oleacein can be observed under this condition.

Overall, our findings well support the efficacy of oleacein in preventing body weight gain and insulin resistance in HFD fed mice. Also, they provide a partial mechanistic molecular explanation of how oleacein may act at the level of the liver, one of the most important target tissues of insulin action. Given that no significant changes were observed in food intake or behavioral signs over the duration of the study, the improvement in insulin resistance in oleacein-treated mice (underlined by the parallel changes in some hepatic molecular targets of insulin), may partially explain the effect of oleacein in preventing weight gain in HFD-fed mice. A minor impact of oleacein was observed when obesity was already established, making it harder to counteract some of the obesity-related metabolic damages. Confirmation of the beneficial effects of oleacein in humans is necessary to consider this compound as an adjuvant to the diet in the prevention and treatment of obesity and obesity-related insulin resistance.

## Ethics Statement

This study was carried out in accordance with the recommendations of the Italian (D.M. 116/92) and ECC regulations (O.J. of E.C.L 358/1 12/18/1986), after approval of the experimental protocol by the institutional ethic committee.

## Author Contributions

GL and SL contributed to animal testing and drafting of the manuscript; CM and VM elaborated figures and tables and contributed to the analysis of the results; CM performed the histopathological analysis; BA and SB performed the molecular analysis; DB, VMM, and NC performed the operation on the animals and supervised the animals’ maintenance during the treatment period; AP and CC performed the preparation of the chemicals and reviewed the final version of the manuscript; AB and DR contributed to the conception of the idea, and critically reviewed and edited the manuscript; MC and NC contributed to animal testing, analysis of the results, and writing of the manuscript. All authors read and approved the submitted version of the manuscript.

## Conflict of Interest Statement

The authors declare that the research was conducted in the absence of any commercial or financial relationships that could be construed as a potential conflict of interest.

## Abbreviations

EVOO, extra-virgin olive oil; HDL, high-density lipoproteins; HFD, high-fat diet; HOMA-IR, homeostatic model assessment of insulin resistance; LDL, low-density lipoproteins; NCD, normocaloric diet.
